# Polyhydroxyalkanoate‐associated phasins as phylogenetically heterogeneous, multipurpose proteins

**DOI:** 10.1111/1751-7915.12718

**Published:** 2017-04-20

**Authors:** Beatriz Maestro, Jesús M. Sanz

**Affiliations:** ^1^ Instituto de Biología Molecular y Celular Universidad Miguel Hernández Av. Universidad s/n Elche 03202 Spain

## Abstract

Polyhydroxyalkanoates (PHAs) are natural polyesters of increasing biotechnological importance that are synthesized by many prokaryotic organisms as carbon and energy storage compounds in limiting growth conditions. PHAs accumulate intracellularly in form of inclusion bodies that are covered with a proteinaceous surface layer (granule‐associated proteins or GAPs) conforming a network‐like surface of structural, metabolic and regulatory polypeptides, and configuring the PHA granules as complex and well‐organized subcellular structures that have been designated as ‘carbonosomes’. GAPs include several enzymes related to PHA metabolism (synthases, depolymerases and hydroxylases) together with the so‐called phasins, an heterogeneous group of small‐size proteins that cover most of the PHA granule and that are devoid of catalytic functions but nevertheless play an essential role in granule structure and PHA metabolism. Structurally, phasins are amphiphilic proteins that shield the hydrophobic polymer from the cytoplasm. Here, we summarize the characteristics of the different phasins identified so far from PHA producer organisms and highlight the diverse opportunities that they offer in the Biotechnology field.

## Introduction

Polyhydroxyalkanoates (PHAs) are natural polyesters produced and accumulated by diverse organisms from the Bacteria and Archaea kingdoms as energy and carbon storage compounds under nutrient limitation conditions (i.e. nitrogen, oxygen or phosphorus) but in the presence of an excess of carbon sources (Anderson and Dawes, 1990; Lee, 1996). These polymers have acquired notoriety in recent years because they display plastic properties similar to their oil‐derived counterparts, but show biodegradability and biocompatibility features which results in a versatile and eco‐friendly alternative (Madison and Huisman, 1999; Potter and Steinbuchel, 2006; Keshavarz and Roy, 2010). PHAs were first described by M. Lemoigne in France, who in the 1920s reported the presence of poly(3‐hydroxybutyrate) [P(3HB)], in the cytoplasm of *Bacillus megaterium* (Lemoigne, 1926). Since then, over 300 species, including both Gram‐positive and Gram‐negative bacteria, have been described with the metabolic ability to synthesize PHAs (Steinbuchel and Fuchtenbusch, 1998; Zinn *et al*., 2001; Suriyamongkol *et al*., 2007; Chanprateep, 2010; Keshavarz and Roy, 2010).

Chemically, PHAs are polyoxoesters of R‐hydroxyalkanoic acid monomers. They are usually classified depending on the number of carbon atoms of the alkyl groups: small chain length PHAs (scl‐PHAs) contain 3–5 carbon atoms [as poly(3‐hydroxybutyrate) ‐P(3HB)‐ or poly(4‐hydroxybutyrate) ‐P(4HB)], whereas medium chain length PHAs (mcl‐PHAs) possess 6–14 carbon atoms [e.g. poly(3‐hydroxyhexanoate), ‐P(3HHx) or poly(3‐hydroxyoctanoate) – P(3HO)]. Long‐chain‐length PHAs (lcl‐PHAs) consisting of hydroxyacids with more than 14 carbon atoms are more scarcely found (Rutherford *et al*., 1995; Singh and Mallick, 2009). These differences are mainly due to the substrate specificity of the PHA synthases from the particular microorganism (Park *et al*., 2012). Moreover, the incorporation of different monomer units in the same chain gives rise to heteropolymers with new properties. The properties and functionalities of the PHAs depend on their monomer composition: whereas scl‐PHAs show thermoplastic properties similar to polypropylene, mcl‐PHAs display elastic features similar to rubber or elastomer (Keshavarz and Roy, 2010; Park *et al*., 2012). Applications of PHAs in the industry are widespread, ranging from the manufacturing of packages and covers to the generation of enantiomeric pure chemicals (Philip *et al*., 2007) or as protein immobilization supports (Draper and Rehm, 2012; Dinjaski and Prieto, 2015; Hay *et al*., 2015). Of significant relevance is the implementation of PHAs in the biomedical discipline, especially supported by the recent FDA approval for P(4HB) to be used as suture material (Tepha Inc., MA, USA). The utility of PHAs in this field arises from their biocompatibility characteristics and has found its application in a variety of processes such as drug delivery, development of medical devices and construction of tissue engineering scaffolds (Misra *et al*., 2006; Wu *et al*., 2009; Wang *et al*., 2010; Xiong *et al*., 2010; Brigham and Sinskey, 2012; Martinez‐Donato *et al*., 2016; Rubio Reyes *et al*., 2016).

**Figure 1:**
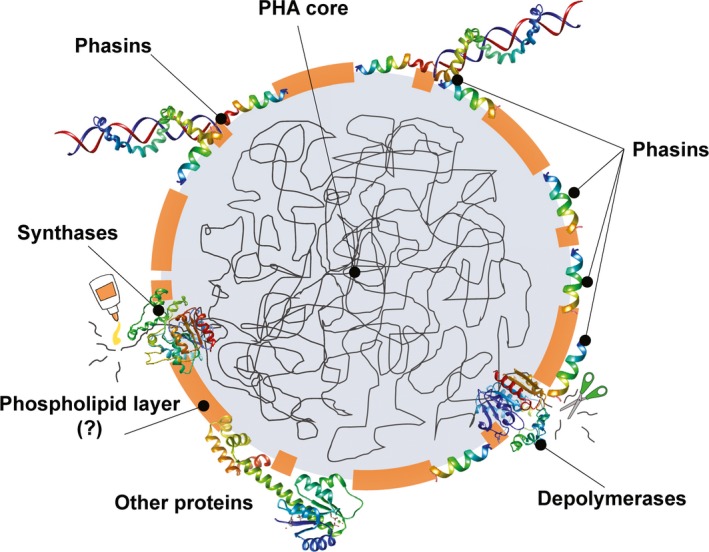
Scheme of the structure of PHA granules.

The PHA polymer accumulates in the cytoplasm in the form of water‐insoluble granules (Fig. 1), the number per cell and size of which depend on the different species and the culture conditions (Jendrossek and Pfeiffer, 2014). Early studies carried out by Merrick′s group showed that these inclusions were constituted by approximately 98% (w/w) PHA, 2% granule‐associated proteins (GAPs) and 0.5% phospholipids (Griebel *et al*., 1968). Since then, several studies have confirmed the presence of a phospholipid layer in PHA preparations (Parlane *et al*., 2016, and references therein). However, some data have put into question the actual presence of the lipid coat *in vivo* (Potter and Steinbuchel, 2006; Beeby *et al*., 2012; Jendrossek and Pfeiffer, 2014), especially from electron cryotomography (Wahl *et al*., 2012) and fluorescence microscopy (Bresan *et al*., 2016) results, according to which the presence of the lipid layer might arise from an experimental artefact on PHA extraction and preparation.

Four different types of GAPs have been identified so far, namely PHA synthases, PHA depolymerases, phasins and other proteins (Steinbuchel *et al*., 1995), the latter including transcriptional regulators as well as hydrolases, reductases and other enzymes involved in the synthesis of PHA monomers (Jendrossek and Pfeiffer, 2014; Sznajder *et al*., 2015). Among them, phasins, which received their name in analogy to oleosins [proteins on the surface of oil globules found in oleaceous plants (Steinbuchel *et al*., 1995)], are the most abundant polypeptides in the PHA carbonosome (Mayer *et al*., 1996). These low molecular weight proteins normally contain a hydrophobic domain, associated with the PHA, and a hydrophilic/amphiphilic domain exposed to the cytoplasm (Potter and Steinbuchel, 2005). On the basis of their sequence, phasins are distributed in four families according to the Pfam database (http://pfam.xfam.org/), namely PF05597, PF09602, PF09650 and PF09361. A recent survey showed that a high percentage of phasins and phasin‐like proteins contains a leucine‐zipper motif in their amino acid sequences, suggesting that oligomerization is a common organization mechanism in these polypeptides (Maestro *et al*., 2013). In the recent years, a large number of phasins have been identified, constituting a phylogenetically heterogeneous group of proteins. We will review the current knowledge on the most representative phasins participating in important biological functions (summarized in Table 1) such as the formation of network‐like covers on the PHA granule surface (Dennis *et al*., 2003, 2008; Pfeiffer and Jendrossek, 2011) or the regulation of the synthesis, morphology, distribution during cell division and degradation of the storage granules (Mezzina and Pettinari, 2016). Finally, the biotechnological potential of this group of proteins will be discussed.

**Table 1:** List of the phasins reviewed in the text, with their most relevant characteristics

Organism	Phasin	Molecular mass (kDa)	UNIPROT accession number (localization)	Most relevant characteristics and roles	References
*Ralstonia eutropha*	PhaP1 _Reu_	20.0	AAC78327 (chromosome 1)	Homotrimer . Major phasin present in *R. eutropha* Plays role in the amount, size and number of granules, and in their degradation . Biotechnological application as immobilization tag	(Steinbuchel *et al*., 1995; Wieczorek *et al*., 1995; York *et al*., 2001a; York *et al*., 2001b; Potter *et al*., 2002; York *et al*., 2002; Potter *et al*., 2004; Banki *et al*., 2005; Barnard *et al*., 2005; Backstrom *et al*., 2007; Kuchta *et al*., 2007; Neumann *et al*., 2008; Wang *et al*., 2008; Yao *et al*., 2008; Chen *et al*., 2014; Sznajder *et al*., 2015)
	PhaP2 _Reu_	20.2	AAP85954 (plasmid pHG1)	Secondary participation in PHB accumulation and mobilization	(Schwartz *et al*., 2003; Potter *et al*., 2004)
	PhaP3 _Reu_	19.6	AY489113 (chromosome 1)	Secondary participation in PHB accumulation and mobilization	(Potter *et al*., 2004)
	PhaP4 _Reu_	20.2	AY489114 (chromosome 2)	Secondary participation in PHB accumulation and mobilization	(Potter *et al*., 2004)
	PhaP5_Reu_	15.7	H16_B1934 (chromosome 2)	Secondary participation in PHB accumulation and mobilization	(Pfeiffer and Jendrossek, 2011)
	PhaP6 _Reu_	22.7	H16_B1988 (chromosome 2)	Secondary participation in PHB accumulation and mobilization	(Pfeiffer and Jendrossek, 2012)
	PhaP7 _Reu_	16.4	H16_B2326 (chromosome 2)	Secondary participation in PHB accumulation and mobilization	(Pfeiffer and Jendrossek, 2012)
*Pseudomonas putida*	PhaF	26.3	Q9Z5E6	Tetramer. Responsible for non‐specific binding to DNA . Intrinsically disordered in its majority unless bound to its ligands . Involved in the PHA biosynthesis, localization of the granules in the cell and in their distribution between daughter cells during cell division . Transcriptional regulator	(Prieto *et al*., 1999; Moldes *et al*., 2004; Ren *et al*., 2010; Galan *et al*., 2011; Dinjaski and Prieto, 2013; Maestro *et al*., 2013)
	PhaI	15.4	Q9Z5E7	Involved in the biosynthesis and accumulation of PHA . Biotechnological application as BioF affinity tag to immobilize or purify fusion proteins	(Prieto *et al*., 1999; Moldes *et al*., 2004; Moldes *et al*., 2006; Ren *et al*., 2010; Dinjaski and Prieto, 2013; Maestro *et al*., 2013)
*Pseudomonas* sp. 61‐3	PhaF	25.6	Q8L3N9	Phasin bound to P(3HB‐co‐3HA) copolymers solely when granules are enriched in 3HA (C6–C12) in more than 13 mol%	(Matsumoto *et al*., 2002 ; Hokamura *et al*., 2015)
	PhaI	15.4	Q8L3P0	Phasin bound to P(3HB‐co‐3HA) copolymers solely when granules are enriched in 3HA (C6–C12) in more than 13 mol%	(Matsumoto *et al*., 2002 ; Hokamura *et al*., 2015)
	PhbP	20.4	A0A0K2QTP6	Phasin bound to P(3HB‐co‐3HA) copolymers solely when granules are enriched in 3HB in more than 87 mol%	(Matsumoto *et al*., 2002 ; Hokamura *et al*., 2015)
*Paracoccus denitrifican*s	PhaP_Pde_	16.5	Q9WX81	Involved in the PHA granule formation, ensuring the correct number and size of granules by preventing coalescence and their distribution throughout the cytoplasm	(Maehara *et al*., 1999)
*Rhodococcus ruber*	GA14	14.2	Q53051 (ORF3)	Binding to the PHA through two hydrophobic patches present in the C‐terminal region of the protein Control of the granule size	(Pieper and Steinbuchel, 1992; Pieper‐Furst *et al*., 1994; Pieper‐Furst *et al*., 1995)
*Azotobacter sp*. FA‐8	PhaP_Az_	20.4	Q8KRE9	Tetramer. PHA binding by amphipathic α‐helices induces protein structuration. Promotes bacterial growth and PHA synthesis. General stress‐reducting action. Chaperone‐like mechanism	(Pettinari *et al*., 2003; de Almeida *et al*., 2007; de Almeida *et al*., 2011; Mezzina *et al*., 2014; Mezzina *et al*., 2015)
*Aeromonas caviae*	PhaP_Ac_	12.6	Q79EN2	Important role in biosynthesis and metabolism of PHA	(Fukui *et al*., 2001; Saika *et al*., 2014; Ushimaru *et al*., 2014; Kawashima *et al*., 2015; Ushimaru *et al*., 2015)
*Aeromonas hydrophila*	PhaP_Ah_	12.6	O32470	Tetrameric in solution, monomeric when bound to PHA granules. Involved in PHA biosynthesis. Controls granule size and number. Transcription regulator of *pha*C gene	(Tian *et al*., 2005; Zhao *et al*., 2016)
*Rhodospirillum rubrum*	ApdA	17.5	Q8GD50	55% identity with Mms16 from *Magnetospirillum*	(Handrick *et al*., 2004a; Handrick *et al*., 2004b)
*Bradyrhizobium diazoefficiens*	PhaP1_Bd_	12.3	Q89JW4	Predominantly alpha‐helical	(Yoshida *et al*., 2013; Quelas *et al*., 2016)
	PhaP2_Bd_	17.3	Q89IS9	Predominantly alpha‐helical	(Yoshida *et al*., 2013; Quelas *et al*., 2016)
	PhaP3_Bd_	12.4	Q89H66	Predominantly alpha‐helical. Minor expression	(Yoshida *et al*., 2013; Quelas *et al*., 2016)
	PhaP4_Bd_	15.4	Q89DP4	Predominantly alpha‐helical. C‐terminal region very rich in alanine residues. Favoured expressed when using yeast extract‐mannitol medium	(Yoshida *et al*., 2013; Quelas *et al*., 2016)

## Phasins from *Ralstonia eutropha*

*Ralstonia eutropha* (formerly *Alcaligenes eutrophus*, and also currently known as *Cupriavidus necator* H16) (Yabuuchi *et al*., 1995) is a Gram‐negative bacterium that produces scl‐PHA and represents the model organism in which biosynthesis and accumulation of poly(3‐hydroxybutyrate) [poly(3HB) or PHB in short], the most commercially successful PHA, has been more thoroughly studied (Sudesh *et al*., 2000; Steinbuchel and Hein, 2001; Stubbe *et al*., 2005; Potter and Steinbuchel, 2006). *Ralstonia eutropha* synthesizes PHB from acetyl‐CoA, catalysed by a β‐ketothiolase (PhaA), an acetoacetyl‐CoA reductase (PhaB) and the key enzyme PHA synthase (PhaC), all three proteins encoded by the *pha*CAB operon (Oeding and Schlegel, 1973; Haywood *et al*., 1988; Schubert *et al*., 1988; Slater *et al*., 1988; Peoples and Sinskey, 1989). The final PHB granules may represent up to 85% of the cell biomass (Vandamme and Coenye, 2004) and are coated with up to seven types of phasins (Potter *et al*., 2004; Pfeiffer and Jendrossek, 2012). Among these, PhaP1_Reu_ is the most abundant one (Sznajder *et al*., 2015) covering an estimated 27–54% of surface of the PHA granules (Tian *et al*., 2005a), and representing around 5% of the total cell protein fraction (Wieczorek *et al*., 1995). PhaP1_Reu_ is only synthesized in PHA‐producing cells in levels correlating well with the PHA accumulation, and it is never found in soluble form but only attached to the granules (Wieczorek *et al*., 1995; York *et al*., 2001a,b, 2002; Tian *et al*., 2005a). Besides PhaP1_Reu_, six additional and minoritaire phasins have also been identified in *R. eutropha* (PhaP2_Reu_‐PhaP7_Reu_). Phasins PhaP2_Reu_‐PhaP4_Reu_ are homologous to PhaP1 and are only synthesized under permissive conditions for PHB accumulation, although in much lower amounts (Potter *et al*., 2004; Pfeiffer and Jendrossek, 2012). On the other hand, the PhaP5_Reu_‐PhaP7_Reu_ proteins are not homologous to PhaP1_Reu_ and probably represent an independent subgroup of phasin‐like proteins. Despite much effort dedicated to this task, the elucidation of the exact role of *R. eutropha* phasins other than PhaP1_Reu_ in PHB homoeostasis remains elusive (Pfeiffer and Jendrossek, 2011, 2012).

Regarding the major phasin PhaP1_Reu_, this polypeptide appears strongly bound to the hydrophobic surface of the PHB polymer as soon as its accumulation starts (York *et al*., 2001b; Cho *et al*., 2012), ensuring the dispersion of the granules and preventing the non‐specific binding of other proteins. PhaP1_Reu_ plays a crucial role in the amount (York *et al*., 2001a,b), size and number of granules (Steinbuchel *et al*., 1995; Wieczorek *et al*., 1995; Kuchta *et al*., 2007) and probably prevents PHB crystallization (Horowitz and Sanders, 1994). It has been demonstrated that PhaP1_Reu_ deletion mutants exhibit less PHB production as compared to the wild‐type strain (Wieczorek *et al*., 1995; York *et al*., 2001b; Kuchta *et al*., 2007), indicating that it is important but not crucial for PHB synthesis, and suggesting that other minor phasins may also contribute to its accumulation. In fact, the expression level of PhaP3_Reu_ significantly increases in PhaP1‐negative mutants (Potter *et al*., 2004). Nevertheless, in the presence of PhaP1 the relative importance of the other phasins must be lower, as the individual deletion of any of them does not induce any appreciable effect on polymer synthesis (Kuchta *et al*., 2007). Moreover, PhaP1_Reu_ deletion mutants only produce a large, single granule per cell unlike wild‐type cells, which usually contain between 6 and 15 disperse, medium‐size granules (Wieczorek *et al*., 1995; Kuchta *et al*., 2007). In contrast, PhaP1_Reu_ overexpression leads to the generation of a high number of small granules (Potter *et al*., 2002).

*Ralstonia eutropha* phasins also play a role in the stability and mobilization of PHB inclusions. Lack of PhaP1_Reu_ in a single deletion mutant causes a certain degree of PHB autodegradation *in vivo*, an event that is dramatically augmented when combined with the multiple deletion of other phasins (Kuchta *et al*., 2007), suggesting that phasins are essential to stabilize the granule. Paradoxically, phasins are also critical for the mobilization of PHB induced by CoA thiolysis as catalysed by the PhaZ depolymerase. While PHB devoid of phasins is unable to be degraded by PhaZ, PhaP1_Reu_ alone is sufficient to assist the depolymerase in PHB degradation (Uchino *et al*., 2007; Eggers and Steinbuchel, 2013). On the other hand, in the absence of PhaP1_Reu_, the other minor phasins may also participate in PHB mobilization to a variable extent (Kuchta *et al*., 2007; Uchino *et al*., 2007; Eggers and Steinbuchel, 2013).

Expression of PhaP1_Reu_ is strictly regulated at the transcription level by PhaR (Potter *et al*., 2002; York *et al*., 2002), thus ensuring that the phasin is produced only when conditions are permissive for PHB accumulation and PhaC is present (York *et al*., 2001a), and in enough quantity to cover all the biopolymer surface, but without inducing a protein stock in the cytoplasm (Wieczorek *et al*., 1995).

It has been proposed that PhaP1_Reu_ possesses a modulatory action on PHB synthesis *in vitro* on a PhaC‐dependent manner. Addition of pure recombinant PhaP1_Reu_ increases the lag phase in the polymer formation for the *R. eutropha* PhaC1 synthase (Cho *et al*., 2012). A two‐hybrid assay did not detect any interaction between the two proteins (Pfeiffer and Jendrossek, 2011). A similar decrease in activity has also been detected for the synthase from *Delftia acidovorans* (PhaC_Da_) (Ushimaru *et al*., 2014) although no mechanism was proposed in this case. On the contrary, PhaP1_Reu_ increases the activity of the synthases from *Aeromonas caviae* (Ushimaru *et al*., 2014) and *Pseudomonas aeruginosa* (Qi *et al*., 2000), this time by reducing the enzymatic lag phase, while it does not affect the activity of PhaC from *Chromatium vinosum* (Jossek *et al*., 1998).

Secondary structure analysis of the PhaP1_Reu_ sequence predicts a highly α‐helical conformation that is characteristic of phasins (Neumann *et al*., 2008). The phasin has been shown to acquire a planar, triangular‐shaped homotrimeric conformation as revealed by small‐angle X‐ray scattering analysis (Neumann *et al*., 2008). First sequence analyses did not unveil a clear, predicted PHA‐binding motif such as long hydrophobic patches (Neumann *et al*., 2008).

## *Pseudomonas* species

Most members of the *Pseudomonas* species are able to accumulate only mcl‐PHA granules based on a well‐conserved gene cluster containing two operons that are transcribed in opposite direction: (i) the *phaC1ZC2D* operon, encoding two type‐II polymerases (PhaC1 and PhaC2), a depolymerase (PhaZ) and the PhaD protein described as a putative transcriptional regulator (Huisman *et al*., 1991; Klinke *et al*., 2000; Steinbuchel and Hein, 2001); and (ii) the *phaFI* operon, located downstream and coding for the PhaF and PhaI phasins (Prieto *et al*., 1999; Sandoval *et al*., 2007).

The mcl‐PHA granules in *Pseudomonas* are covered by a protein layer that contains the PhaF and PhaI phasins, together with PhaC, PhaZ and the acyl‐CoA synthetase ACS1 (Prieto *et al*., 1999; Moldes *et al*., 2004; Peters and Rehm, 2005; de Eugenio *et al*., 2007; Sandoval *et al*., 2007; Ruth *et al*., 2008).

**Figure 2:**
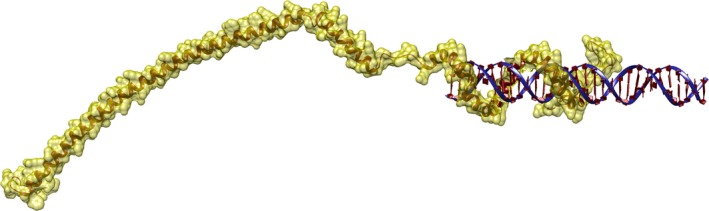
Predicted structure of a monomer of the PhaF phasin from *Pseudomonas putida* KT2440 complexed to DNA (Maestro *et al*., 2013).

PhaF is the major phasin in *Pseudomonas* species*,* and it is structurally organized in two well‐defined domains (Prieto *et al*., 1999; Moldes *et al*., 2004), (i) the *N*‐terminal, PHA‐binding domain, (referred to as BioF in the case of *P. putida* GPo1), which shares sequence similarity with PhaI, and (ii) the C‐terminal moiety, a highly positively charged, histone‐like domain, containing eight AAKP‐like tandem repeats, and responsible for non‐specific binding to DNA (Prieto *et al*., 1999; Moldes *et al*., 2004; Galan *et al*., 2011). Biophysical studies carried out on PhaF, supported by a three‐dimensional structural model, suggest an elongated disposition in which the PHA‐binding domain acquires an amphipathic helix conformation suitable to recognize the surface of the polymer granule and that is separated from the DNA‐binding domain by a short leucine zipper presumably involved in the protein tetramerization (Maestro *et al*., 2013) (Fig. 2). Remarkably, similar coiled‐coil sequences were found in the majority of phasins included in the UniProtKB database, suggesting that oligomerization might constitute a common feature of these proteins (Maestro *et al*., 2013). Moreover, the protein might be intrinsically disordered in its majority unless bound to its ligands (PHA and DNA), a trait that is also probably shared by many other phasins (Maestro *et al*., 2013).

The functionality of PhaF is not only ascribed to a mere role in PHA intracellular stabilization, but it also plays a critical role in the localization of the granule in the cell centre, ensuring an equal distribution between daughter cells during cell division by a simultaneous attachment to the PHA polymer and to nucleoid DNA (Galan *et al*., 2011; Maestro *et al*., 2013). In this sense, lack of PhaF induces *in vivo* a considerable reduction in total PHA content as the defects caused in granule segregation gives rise to population heterogeneity (Galan *et al*., 2011; Dinjaski and Prieto, 2013). Interestingly, a similar function has been detected for the PhaM protein in *R. eutropha*, a phasin‐like polypeptide responsible for attachment of PHB granules to the bacterial nucleoid, ensuring an almost equal number of PHB granules to that both daughter cells after cell division (Pfeiffer *et al*., 2011; Wahl *et al*., 2012). Finally, it has been demonstrated that PhaF is also involved in the in the control of expression of the *pha*C1 synthase and *pha*I phasin genes (Prieto *et al*., 1999; Galan *et al*., 2011).

The PhaI phasin displays a high sequence similarity with the PHA‐binding domain of PhaF, including the probable Leu‐zipper sequence. Together with PhaF, it has been demonstrated to be essential for optimal PHA biosynthesis and accumulation in *P. putida* KT2442 and *P. putida* U (Ren *et al*., 2010; Dinjaski and Prieto, 2013) although it can be replaced by the homologous PHA‐binding domain of PhaF (Dinjaski and Prieto, 2013).

While most *Pseudomonas spp* accumulate only mcl‐PHA, some strains such as *Pseudomonas* sp.61‐3, *Pseudomonas* sp14‐3 and *P. pseudoalcaligenes* are also able to accumulate scl‐PHA such as PHB. In these cases, an additional *phb* cluster has been identified, containing genes coding for the proteins PHB synthase (PhbC), β‐ketothiolase (PhbA), NADPH‐dependent acetoacetyl coenzyme A reductase (PhbB) and the PhbP phasin involved in scl‐PHA metabolism (Matsusaki *et al*., 1998; Ayub *et al*., 2007; Manso Cobos *et al*., 2015). Interestingly, in *Pseudomonas* sp.61‐3 it has been demonstrated a certain degree of PHA specificity by the phasins, as PhaF and PhaI appear bound to P(3HB‐co‐3HA) copolymers only when the 3HA (C6–C12) composition is present in more than 13 mol %, whereas PhbP is solely found in 3HB enriched granules in more than 87 mol% (Hokamura *et al*., 2015).

## *Paracoccus denitrificans*

*Paracoccus denitrificans* is a facultative methylotrophic bacterium capable of synthesizing scl‐PHAs from several alcohols (Yamane *et al*., 1996). The major phasin associated with PHA granules in *P. denitrificans* is PhaP_Pde_ (GA‐16) (Maehara *et al*., 1999). The expression of the *pha*P gene is negatively controlled by the auto‐regulated repressor PhaR (Maehara *et al*., 2002), and a positive correlation between the accumulation of PhaP_Pde_ protein and production of PHA has been demonstrated (Maehara *et al*., 1999). PhaP_Pde_ plays a structural role in the PHA granule formation, constituting an amphipathic layer, preventing the coalescence of the granules and ensuring the correct number and size of granules. Besides, it is also involved in the distribution of the granules throughout the cytoplasm (Maehara *et al*., 1999).

## *Rhodococcus ruber*

The coryneform bacterium *Rhodococcus ruber* NCIMB 40126 accumulates a copolyester of 3‐hydroxybutyric acid and 3‐hydroxyvaleric acid from single, unrelated carbon sources (Haywood *et al*., 1991). The GA14 protein has been identified as the major phasin bound to the surface of the PHA granules, showing a direct correlation between the amount of protein and the level of PHA synthesis in the cells (Pieper and Steinbuchel, 1992; Pieper‐Furst *et al*., 1994). The C‐terminal region of the protein, containing two hydrophobic patches, has been demonstrated as responsible for the granule anchoring (Pieper‐Furst *et al*., 1995). This protein has also been isolated from lipid inclusions in this bacterium (Kalscheuer *et al*., 2001).

## Azotobacter genus

PhaP_Az_ is the most abundant PHB granule‐associated protein observed in *Azotobacter sp*. FA‐8 (Pettinari *et al*., 2003; Mezzina *et al*., 2015). This protein displays a growth‐promoting effect, also enhancing the polymer production in recombinant PHB‐producing *Escherichia coli* (de Almeida *et al*., 2007, 2011). Moreover, it exerts a stress‐reduction action, both in PHB and non‐PHB synthesizing bacteria, decreasing the induction of heat shock‐related genes in *E. coli* (de Almeida *et al*., 2011) and promoting protein folding through a chaperone‐like mechanism, which suggests an *in vivo* general protective role of this phasin (Mezzina *et al*., 2015).

PhaP_Az_ has been suggested to conform a coiled‐coil tetramer when it is not bound to any target. Secondary structure analysis predicts the existence of α‐helices and disordered regions, with two amphipathic helices probably responsible for protein‐protein or PHB interactions. Spectroscopical studies suggest that hydrophobic environments, such as those provided by PHB, can induce phasin structuration (Mezzina *et al*., 2014).

## *Aeromonas* genus

*Aeromonas caviae* FA440 is a Gram‐negative bacterium isolated from soil that is capable of producing copolyesters consisting of scl‐ and mcl‐PHA from alkanoates or oils (Doi *et al*., 1995). This organism possesses a biotechnological potential as the films made of the random copolymer of (R)‐3‐hydroxybutyrate and (R)‐3‐hydroxyhexanoate [P(3HB‐co‐3HHx)] produced by this bacteria have demonstrated very good soft and flexible properties, and better biocompatibility when compared to a P(3HB) homo‐polymer, making them suitable for more practical applications (Doi *et al*., 1995; Yang *et al*., 2002). The PHA biosynthetic operon in *A. caviae* consists on *pha*P‐*pha*C‐*pha*J genes, which encode the PHA granule‐associated protein phasin (PhaP_Ac_) (Fukui *et al*., 2001), as well as the PhaC_Ac_ synthase (Fukui and Doi, 1997), and the R‐specific enoyl‐CoA hydratase (PhaJ_Ac_) (Fukui *et al*., 1998).

The PHA granules isolated from *A. caviae* are relatively simple in terms of its GAPs composition, as their protein cover only comprises the PHA synthase and the PhaP_Ac_ phasin (Fukui *et al*., 2001). PhaP_Ac_ (also referred to as GA13) is a 13‐kDa protein, which shows an appreciable similarity with the PhaP phasin from *Acinetobacter sp*. (Fukui *et al*., 2001). Moreover, no hydrophobic or amphiphilic regions are evident in the primary structure of this protein (Fukui *et al*., 2001).

PhaP_Ac_ plays an important role in the biosynthesis and metabolism of PHAs. A high level activity of PHA synthase has been documented when overexpression of *pha*C_Ac_ takes place together with *pha*P_Ac_, and the PHA copolymer composition is enriched in the 3HHx fraction when compared to overexpression of *pha*C_Ac_ alone, although the substrate specificity of PhaC_Ac_ is not affected in this conditions (Fukui *et al*., 2001). Besides, in a recombinant strain of *R. eutropha* which is capable of synthesizing P(3HB‐co‐3HHx), the replacement of the PhaP1_Reu_ phasin by PhaP_Ac_ resulted in an increase in 3HHx proportion in the copolymer (Kawashima *et al*., 2015). Moreover, the activity of PhaC_Ac_ synthase *in vitro* is activated by the presence of PhaP_Ac_ both in the prepolymerization and the polymer‐elongation states, and the *in vivo* P(3HB) accumulation in a recombinant *E. coli* strain expressing PhaP_Ac_ increased 2.3‐fold when compared with the corresponding PhaP_Ac_‐free strain (Ushimaru *et al*., 2014). This effect is not due to a mere increase in the amount of soluble PhaC_Ac_, but probably arises from the phasin assisting the withdrawal of the growing PHA polymer chain from PhaC_Ac_ (Ushimaru *et al*., 2014). In contrast, the prepolymerization activities of PhaC_Re_ and PhaC_Da_ synthases decrease by the presence of PhaP_Ac_, whereas the activity of polymer‐elongating PhaC_Re_ is not affected. Interestingly, the *in vivo* accumulation of P(3HB) increases 1.2‐fold in a recombinant *E. coli* strain when PhaP_Ac_ is expressed together with PhaC_Re_, compared to the phasin‐free strain. As the amount of PhaC_Re_ in the soluble fraction increases approximately threefold by PhaP_Ac_ coexpression, this has led to postulate that this enhanced PHA accumulation could be attributed to a chaperone‐like role of PhaP_Ac_ in the folding of PhaC_Re_ (Ushimaru *et al*., 2014). Finally, an enhancement in the *in vivo* PHA accumulation has been observed in *E. coli* harbouring the *pha*PCJ operon from *A. caviae* when a single nucleotide mutation is present in the *pha*P_Ac_ gene (PhaP_Ac_D4N) (Saika *et al*., 2014). The mutation does not induce an increase in the activity of the PHA synthase, but a higher expression level of *pha*P_Ac_ gene was demonstrated, suggesting that this effect could be attributed to the enhanced expression of the whole *pha*PCJ operon (Ushimaru *et al*., 2015).

Another *Aeromonas* species, *A. hydrophila* 4AK4, is a Gram‐negative bacterium initially isolated from raw sewage samples that is able to accumulate 35–50 wt. % copolymer [P(3HB‐co‐3HHx)] (Lee *et al*., 2000) reaching 70 wt. % in a metabolic engineered strain (Qiu *et al*., 2006; Liu *et al*., 2011), so this microorganism has been used for the industrial‐scale production of this PHA (Chen *et al*., 2001). A *pha* operon similar to *A. caviae* has been found in this species (Qiu *et al*., 2006). The phasin produced by this microorganism (PhaP_Ah_) is a 13‐kDa protein whose overexpression leads to a higher number and a decrease in size of P(3HB‐co‐3HHx) granules, as well as to an increase in *pha*C_Ah_ gene transcription and to an increment of 3HHx fraction on the P(3HB‐co‐3HHx) accumulated copolymer, concomitantly with a reduced molecular weight of the polyester (Tian *et al*., 2005b). The 3‐D structure of PhaP_Ah_ has been recently elucidated by X‐ray crystallography (Zhao *et al*., 2016). The protein folds in solution into a brick‐like tetramer built from the packing of four amphipathic α‐helical monomers through their corresponding hydrophobic faces. On the basis of several biophysical and mutational studies, it has been suggested that in the presence of hydrophobic entities such as PHB surfaces, the tetramer dissociates and individual monomers are able then to interact with the non‐polar compound (Zhao *et al*., 2016).

## *Rhodospirillum rubrum*

*Rhodospirillum rubrum* is a Gram‐negative, phototrophic, purple, non‐sulfur bacterium with a huge metabolic flexibility that allows it to produce many different types of storage polyesters, such as PHB, the poly‐(3‐hydroxybutyrate‐co‐3‐hydroxyvalerate) [P(3HB‐co‐3HHx)] copolymer, or even more polymers including β‐hydroxyhexanoate or β‐hydroxyheptanoate monomers, depending on the carbon source (Brandl *et al*., 1989). This organism appears well suited for fermenting synthesis gas raw materials, making it especially attractive for the bioconversion of syngas feedstocks into [P(3HB‐co‐3HHx)] copolyester (Do *et al*., 2007; Revelles *et al*., 2016).

ApdA (activator of polymer degradation) is a 17.5‐kDa phasin that is bound to the PHB granules *in vivo* in *R. rubrum* (Handrick *et al*., 2004a). It is absolutely required for the efficient hydrolysis *in vitro* of the native PHB (nPHB) granules by the PhaZ1 depolymerase, a role that is not affected by several physical and chemical stresses, such as high temperatures, extreme pH's or 5 M guanidinium, but that can be mimicked by the pretreatment of the granules with trypsin or other proteases, although no protease activity has been found for this phasin (Handrick *et al*., 2004a,2004b). On the other hand, ApdA presents a 55% identity with Mms16, a magnetosome‐associated protein in *Magnetospirillum* that has also been shown, in turn, to act as a phasin‐like protein bound to the PHB granules produced by this bacteria (Handrick *et al*., 2004a; Schultheiss *et al*., 2005). In fact, it has been shown that Mms16 is able to functionally replace the activating role of ApdA in *R. rubrum* (Handrick *et al*., 2004a).

## *Bradyrhizobium diazoefficiens*

*Bradyrhizobium diazoefficiens* is a Gram‐negative soil bacterium that accumulates a large amount of PHB, a process that competes with the fixation of atmospheric N_2_ in symbiosis with soybean plants (Romanov *et al*., 1980). Four phasins have been identified in PHA granules from *B. diazoefficiens*, namely PhaP1_Bd_‐PhaP4_Bd_ (Yoshida *et al*., 2013). None of them are involved in the bacterial growth kinetics (Quelas *et al*., 2016), but they are all expressed in levels that correlate with the accumulated PHA (Yoshida *et al*., 2013). In any case, expression of PhaP4_Bd_ is favoured when using yeast extract‐mannitol (YM) medium, and it presents the highest affinity to PHA granules *in vitro* (Yoshida *et al*., 2013). Transcription of *phaP*3 seems to be low and constant during growth, suggesting that this phasin does not have a relevant role in PHA metabolism (Yoshida *et al*., 2013). On the other hand, the study of single and double mutants has revealed that the combined role of PhaP1_Bd_ and PhaP4_Bd_ must be crucial in determining the number and size of the granules (Quelas *et al*., 2016).

Structurally, PhaP1_Bd_‐PhaP4_Bd_ are predicted to be predominantly alpha‐helical but only PhaP4_Bd_ contains additionally a C‐terminal region very rich in alanine residues (13 out of 34 amino acids) (Yoshida *et al*., 2013). Besides, they are all proposed to oligomerize (Quelas *et al*., 2016).

## Other phasins

Several other phasin proteins have been identified in other organisms such as *Sinorhizobium meliloti*,* Haloferax mediterranii* or *Herbaspirillum seropedicae*, but there is little information about them other than their involvement in PHA accumulation (Wang *et al*., 2007; Cai *et al*., 2012; Tirapelle *et al*., 2013; Alves *et al*., 2016).

## Binding of phasins to PHA

**Figure 3:**
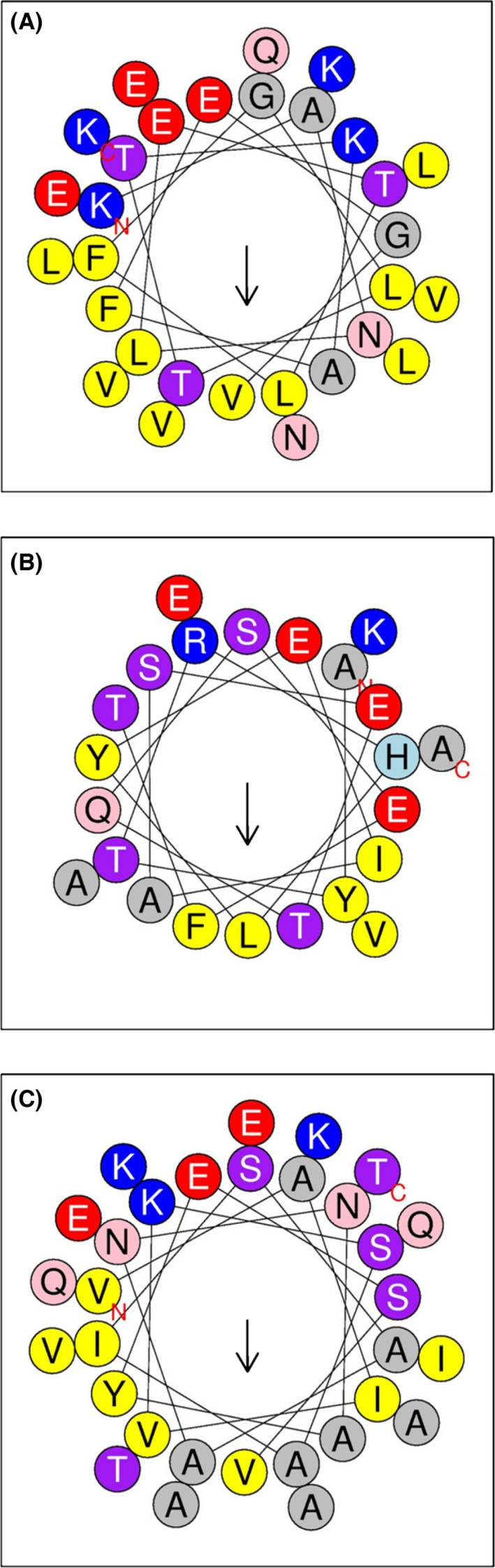
HeliQuest prediction of amphipathic α‐helices in the sequence of PhaP1_Reu_ from *R. eutropha*, belonging to Pfam family PF05597. *A. residues 13–42 (Mean hydrophobic moment ‐arrow‐) <μH> = 0.39); * *B. residues 81–103 (<μH> = 0.40);* C. residues 131–161 (<μH> = 0.34). See Fig. S1 for details.

Little is known about the molecular details of phasin‐PHA interaction. In the absence of deeper biophysical analyses, some speculations can be made on the basis of the scarce protein structural data and secondary structure predictions. As described above, it has been suggested for the *P. putida* KT2442 PhaF phasin a non‐specific interaction through an amphipathic α‐helix, so that the hydrophobic side of the helix faces the polymer whereas the hydrophilic side is exposed to the solvent. Such statement is based on the fact that the granule‐binding sequence also interacts strongly with hydrophobic compounds (oleic acid) and chromatographic resins (phenyl‐sepharose) (Maestro *et al*., 2013). This idea receives considerable support after the elucidation of the PhaP_Ac_ three‐dimensional structure (Zhao *et al*., 2016), which confirms the widespread presence of amphipathic sequences along this protein. In addition, selected mutants of PhaP_Ac_ designed to increase the amphipathic character of the helices concomitantly led to a stronger binding to P(3HB‐co‐3HHx) films (Zhao *et al*., 2016). With the aim of checking whether this proposed mechanism might represent a common procedure used by phasins to interact with the PHB granule, we have carried out a theoretical study of secondary structure and amphipathicity prediction for each of the four Pfam phasin families. Due to the high number of phasin sequences to be analysed, we generated a consensus sequence for each family using the Jalview utility (Waterhouse *et al*., 2009). Then, a secondary structure prediction was carried out for each consensus sequence using Jpred4 (Drozdetskiy *et al*., 2015), and finally, all predicted α‐helical sequences were analysed for their amphipathicity with HeliQuest (Gautier *et al*., 2008). The results show phasins (belonging to the four Pfam families) as generally predicted, highly helical proteins with appreciable amphipathic stretches (See Fig. S1 and Fig. 3 for the specific case of PhaP1_Reu_ from *R. eutropha*). This simple theoretical model, in the absence of more experimental confirmation, would explain some observations such as the lack of a defined PHA‐binding region in PhaP1_Reu_, as the PHA‐binding ability seems distributed throughout the protein (Neumann *et al*., 2008).

## Biotechnological application of phasins

The amphiphilic character of phasins makes them suitable to be used as natural biosurfactants. In this sense, pure recombinant PhaP_Ah_ from *A. hydrophila* 4AK4 shows a strong effect to form emulsions with lubricating oil, diesel and soybean oil when compared with bovine serum albumin, sodium dodecylsulfate, Tween 20 or sodium oleate, even retaining its activity after heat‐treatment of the protein or the emulsions themselves (Wei *et al*., 2011).

In any case, the most widely studied application of phasins arises from their PHA‐binding capacity. In this regard, the *N*‐terminal, PHA‐binding domain of PhaF from *Pseudomonas putida* GPo1 (referred to as BioF sequence) has shown to be very effective as an affinity tag to immobilize *in vivo* fusion proteins using mcl‐PHA as support (Moldes *et al*., 2004, 2006). Polyester granules carrying BioF‐tagged fusion proteins can be easily isolated by centrifugation and used directly or, if required, the purification of the adsorbed protein can be achieved by gentle elution with detergents, keeping their full activity in both cases (Moldes *et al*., 2004). This system has been demonstrated to be an eco‐friendly way to deliver active proteins to the environment such as the Cry1Ab toxin with insecticidal activity (Moldes *et al*., 2006).

Similar *in vivo* immobilization procedures have also been developed for PhaP1_Reu_ using *E. coli* as heterologous host for the PHA synthesis (Chen *et al*., 2014). In this case, the gene coding for the D‐hydantoinase (D‐HDT) (enzyme involved in the generation of D‐amino acids of commercial values such as one of the precursors required for the synthesis of semi‐synthetic antibiotics) was fused to *pha*P1. The recombinant fusion protein, PhaP1_Reu_‐HDT, resulted to be effectively attached to the granules, and the enzyme showed to be active and stable (Chen *et al*., 2014). In a further development, Wood′s group also used the PhaP1_Reu_ phasin and *E. coli* or *R. eutropha* as expression and immobilization hosts, but in this case they incorporated a self‐cleaving intein sequence between the affinity tag and the protein of interest, allowing the easy removal of the tag and the subsequent purification of the native product by a simple pH change (Banki *et al*., 2005; Barnard *et al*., 2005). The advantage of these procedures comes from the fact that both protein and support are easily and effectively produced by the same bacterial host, leading to cost reduction in the downstream process. In any case, binding and purification can also be carried out *in vitro*, allowing protein production in a continuous way as demonstrated by Wang and coworkers for PhaP_Ac_ (Wang *et al*., 2008).

The specific immobilization of fusion proteins to PHA *via* phasins is starting to be employed in medicine, both in diagnostic and drug delivery applications. In the first case, two hybrid genes encoding either the mouse interleukin‐2 (IL2) or the myelinoligodendrocyte glycoprotein (MOG) fused to PhaP1_Reu_ were constructed and expressed in a recombinant, PHA‐accumulating *E. coli* strain. The PHA beads obtained from this system displayed the eukaryotic proteins correctly folded, and they were subsequently implemented for specific and sensitive antibody detection using the fluorescence‐activated cell sorting (FACS) technology (Backstrom *et al*., 2007). In another example, two recombinant fusion proteins with PhaP1_Reu_ were generated to achieve specific delivery: mannosylated human α1‐acid glycoprotein (hAGP), that is able to bind to the mannose receptor of macrophages, and a human epidermal growth factor (hEGF), able to recognize EGF receptors on carcinoma cells. The resulting proteins (rhAGP–PhaP1_Reu_ and rhEGF–PhaP1_Reu_) were self‐assembled on P(3HB‐co‐3HHx) nanoparticles, achieving the specific delivery of the payload both *in vitro* and *in vivo* (Yao *et al*., 2008). On the other hand, the sequence coding for a peptide containing the amino acids Arg‐Gly‐Asp, the most effective peptide sequence used to improve cell adhesion on artificial surfaces, was fused to PhaP_Ac_ (Dong *et al*., 2010). Different polyesters, such as P(3HB‐co‐3HHx) or P(3HB‐co‐3HV), were coated with purified PhaP‐RGD hybrid protein, and the complex proved effective in adhesion and improvement of cell growth on two different fibroblast cellular lines, suggesting viable applications on implant biomaterials (Dong *et al*., 2010).

## Concluding remarks

The generic name of ‘phasin’ denotes a set of proteins which indeed share the ability to recognize and adsorb to PHA polyesters. They play an essential contribution in the physical stabilization of the PHA granule within the cell, ensure the correct distribution of the polyester upon cell division and assist other proteins (synthases and depolymerases) on PHA metabolism. Nevertheless, their specific role is highly dependent both on the microbial strain and on the metabolic state of the cell. Their versatility is such that they may even participate in opposite events (e.g. synthesis and degradation of the PHA polymer). Besides, their strong affinity to PHA allows their use as protein affinity tags for polymer functionalization and therefore constitutes an opportunity to develop valuable applications in biotechnology and biomedicine. Although little structural data are still available, phasins are predicted to acquire relatively simple, amphipathic, three‐dimensional structures and to bind to PHA *via* non‐specific hydrophobic interactions. This makes them amenable to be easily engineered to produce recombinant variants that display a modulated affinity to PHA, that may be useful both for *in vivo* PHA production and *in vitro* biotechnological and biomedical applications.

## Conflict of interest

None declared.

## Supporting information


**Fig. S1.** (A–D). Secondary structure and amphipatic α‐helix predictions of consensus sequences derived from phasin‐related Pfam families (http://pfam.xfam.org/).
