# Supplementary material for: Polyhydroxyalkanoate‐associated phasins as phylogenetically heterogeneous, multipurpose proteins
Source: Microb Biotechnol. 2017 Apr 20;10(6):1323–37. doi: 10.1111/1751-7915.12718 (PMC5658603; doi:10.1111/1751-7915.12718)
Supplement: Supplementary file 1 — Fig. S1. (A–D). Secondary structure and amphipatic α‐helix predictions of consensus sequences derived from phasin‐related Pfam families (http://pfam.xfam.org/). [file MBT2-10-1323-s001.pdf]

A) **Pfam family PF05597 -** 36 seed sequences (264 sequences in total)

JalView consensus sequence and JPred secondary structure prediction:

```
1                                                                    70
KKQASQLAGEVKDSARQIWLAGLGAFAKAQEEGGKLFEALVKEGESLEKGQSGDKTQKVAEEQLEAARAG
----HHHHHHHHHHHHHHHHHHHHHHHHHHHHHHHHHHHHHHHHHHHHHHHHHHHHHHHHHHHHHHHHHH

71                                                                   140
VGSQADRVKSMVSDVKAKAEGQWDRFGSKLEKAFDERVAKALNRLGVPSRKDVEALSARVDELTAELERL
HHHHHHHHHHHHHHHHHHHHHHHHHHHHHHHHHHHHHHHHHHH------HHHHHHHHHHHHHHHHHHHHH

 142
AG
--
```

Subsequence-1 (residues 21-50)
Mean hydrophobic moment <μH> = 0.39

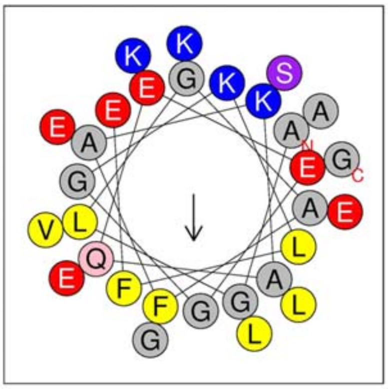

Subsequence-2 (residues 76-113)
Mean hydrophobic moment <μH> = 0.45

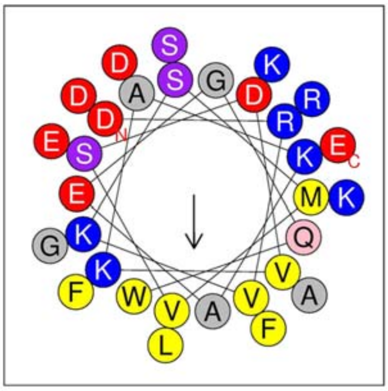

Subsequence-3 (residues 120-140)
Mean hydrophobic moment <μH> = 0.48

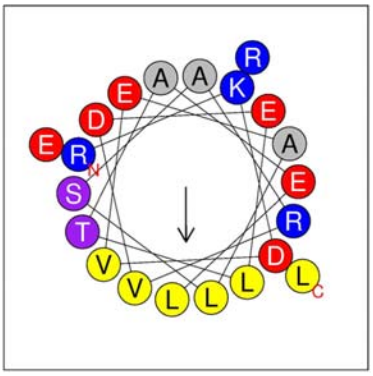

B) **Pfam family PF09602 -** 4 seed sequences (18 sequences in total)

JalView consensus sequence and JPred secondary structure prediction:

```
1                                                                    70
METKAYELVVDAFWDQWEESLELFASAGKQIEQATLKALEQQQDAIHEFTKALSELEAEDKQFTAEFEDL
-----HHHHHHHHH-HHHHHHHHHHHHHHHHHHHHHHHHHHHHHHHHHHHHHHHHHHHHHHHHHHHHHHH

71                                                                  140
LQKTVENLASEAGNADADNIEEWTDKLEEASAKFDELALSPSKSSFDLLDQAEEQFEETLKQFIEQQQKN
HHHHHHHHH-----HHHHHHHHHHHHHHHHHHHHHHHHHHHHHHHHHHHHHHHHHHHHHHHHHHHHHHHH

141                      166
REEAQKVLEAYVEQAKDSQKELAKKFEE
HHHHHHHHHHHHHHHHHHHHHHHHH---
```

Subsequence-1 (residues 6-79)
Mean hydrophobic moment <μH> = 0.43
(Figure limited to the first 54 residues)

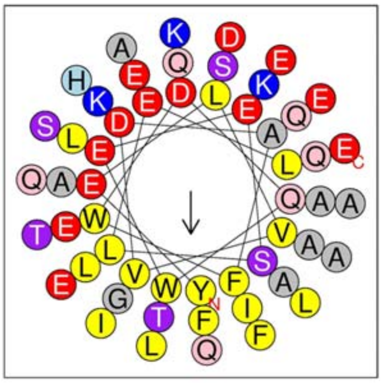

Subsequence-2 (residues 87-110)
Mean hydrophobic moment <μH> = 0.48

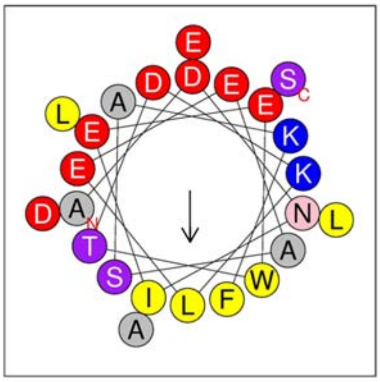

Subsequence-3 (residues 116-167)
Mean hydrophobic moment <μH> = 0.43

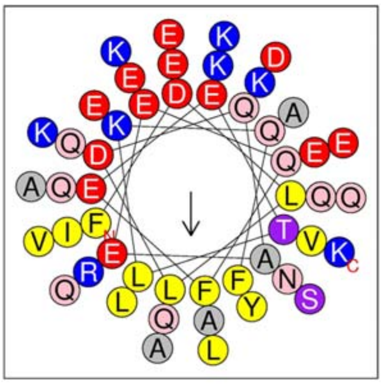

C) **Pfam family PF09650 -** 47 seed sequences (173 sequences in total)

JalView consensus sequence and JPred secondary structure prediction:

```
1                                                                    70
IHIEREHSLGLAEAREAAEKLAEKLEEEFGGKVAAGLECTWEEGETGDTLRFKRSGAMGQGVDGTLEVGP
-EEEE-----HHHHHHHHHHHHHHHHHHH------EEEEEEE------EEEEEEEEE----EEEEEEE--
```

```
71                                104
DKVRLDAKLGFLLWRSAFKGRIESEIEKNLDKLL
-EEEEEEE--HHHHHHH-HHHHHHHHHHHHHH--
```

Subsequence-1 (residues 11-29)
Mean hydrophobic moment <μH> = 0.45

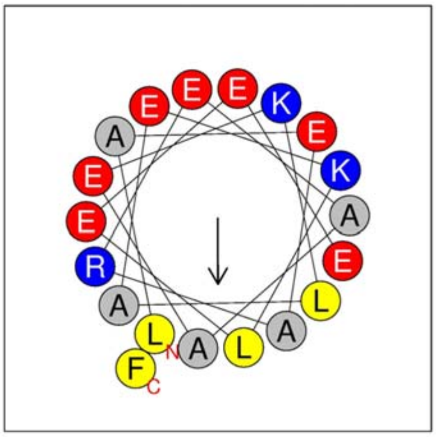

D) **Pfam family PF09361 -** 246 seed sequences (789 sequences in total)

JalView consensus sequence and JPred secondary structure prediction:

1 70
EQFAAAANKANLEAQLALLVALASLAFKGAEKLAELNLEAAKASLEESVAAAKALLGACKGDVESPQELF
---HHHHHHHHHHHHHHHHHHHHHHHHHHHHHHHHHHHHHHHHHHHHHHHHHHHHHH--------HHHHH

71 111
DELQSELAQPAAQAEKAVAYARKLYELAAKTQAEAAKPAEAQV
HHHHHHHH--HHHHHHHHHHHHHHHHHHHHHHHHHHHHHHH--

Subsequence-1 (residues 13-33)
Mean hydrophobic moment <μH> = 0.33

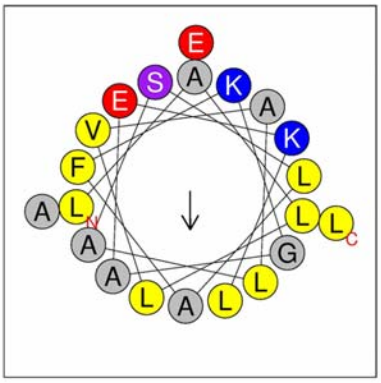

Subsequence-2 (residues 67-107)
Mean hydrophobic moment <μH> = 0.34

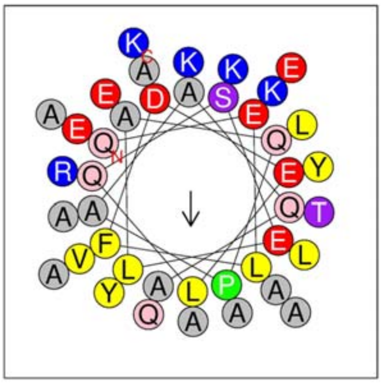

Figure S1 (A-D). Secondary structure and amphipatic α-helix predictions of consensus sequences derived from phasin-related Pfam families (http://pfam.xfam.org/). Seed sequences extracted from each Pfam family were subjected to Jalview analysis (http://www.jalview.org/) to obtain a consensus sequence which was subsequently subjected to secondary structure prediction with the Jpred4 utilities (http://www.compbio.dundee.ac.uk/jpred/). *H* and *E* represent residues in α-helix and extended (β) comformations, respectively. All predicted α-helical sequences were then analyzed for they amphipathicity with HeliQuest (http://heliquest.ipmc.cnrs.fr/). Helical wheels are depicted for those subsequences with a mean hydrophobic moment (<μH>, symbolized by an arrow) higher than 0.30, representing a relevant amphipathic character.
